# A Novel, Controllable, and Efficient Method for Building Highly Hydrophobic Aerogels

**DOI:** 10.3390/gels10020121

**Published:** 2024-02-02

**Authors:** Shu-Liang Li, Yu-Tao Wang, Shi-Jun Zhang, Ming-Ze Sun, Jie Li, Li-Qiu Chu, Chen-Xi Hu, Yi-Lun Huang, Da-Li Gao, David A. Schiraldi

**Affiliations:** 1 SINOPEC (Beijing) Research Institute of Chemical Industry Co., Ltd., 14 Beisanhuan East Road, Chaoyang District, Beijing 100013, China; lishl.bjhy@sinopec.com (S.-L.L.); zhangsj.bjhy@sinopec.com (S.-J.Z.); lijie.bjhy@sinopec.com (J.L.); chulq.bjhy@sinopec.com (L.-Q.C.); hucx.bjhy@sinopec.com (C.-X.H.); huangyl.bjhy@sinopec.com (Y.-L.H.); 2 Department of Macromolecular Science and Engineering, Case Western Reserve University, Cleveland, OH 44106-7202, USA; mxs1031@case.edu (M.-Z.S.); das44@case.edu (D.A.S.)

**Keywords:** thermal insulation, hydrophobic, aerogel, freeze-drying, template method

## Abstract

Aerogels prepared using freeze-drying methods have the potential to be insulation materials or absorbents in the fields of industry, architecture, agriculture, etc., for their low heat conductivity, high specific area, low density, degradability, and low cost. However, their native, poor water resistance caused by the hydrophilicity of their polymer matrix limits their practical application. In this work, a novel, controllable, and efficient templating method was utilized to construct a highly hydrophobic surface for freeze-drying aerogels. The influence of templates on the macroscopic morphology and hydrophobic properties of materials was investigated in detail. This method provided the economical and rapid preparation of a water-resistant aerogel made from polyvinyl alcohol (PVA) and montmorillonite (MMT), putting forward a new direction for the research and development of new, environmentally friendly materials.

## 1. Introduction

Aerogels made from silane [1,2,3,4,5], carbon [6,7,8,9], biomass [10,11,12,13,14,15,16,17,18,19,20,21,22,23], polymers [24,25,26], metal oxides [27], inorganic salts [28,29], etc., have the advantages of low density, high porosity, and high specific area [18]. This combination of properties makes them have a huge number of applications in the fields of industry [30], architecture [8,31,32], agriculture [17], etc., some of which have been commercialized already. Among them, aerogels made from a soluble polymer matrix by freeze-drying have the potential to be applied to thermal or sound insulation because of their low cost, easy preparation, and low heat conductivity [1,9,30]. However, the skeletons of these aerogels have hydroxyl or other polar groups, which make them prone to absorbing water vapor in moist atmospheres [5,12,33]. This usually results in a reduction in the mechanical properties and insulation of aerogels [2]. What is worse is that the skeletons will degrade quicker when they are wet over a long time period, shortening their operational life. Moreover, when water droplets condense on or are absorbed by the porous structure, the skeleton of the aerogel will be destroyed by capillary force and collapse [24]. Therefore, hydrophobic modification is a necessary precondition for the practical application of aerogels [15,31].

The current hydrophobic modifications of porous materials can be classified into integral coating and skeleton treatment based on the treatment scales [14,32]. The former treatment involves directly coating the hydrophobic layer on the block of porous materials. It usually causes dimensional changes and the destruction of surficial porous structures. The latter treatment utilizes physical or chemical methods to endow the surface of the skeleton with hydrophobicity [34]. Chemical vapor deposition (CVD) is a feasible method, with the obvious advantages of effect and size retention, but it cannot rebuild rough morphology. In fact, according to the Wenzel model, the hydrophobicity of the surface is highly dependent on the roughness [18,21]. Adding or coating nano–micro-materials is an effective method, but it complicates the process and increases the cost. Therefore, an inexpensive and effective method is required to build highly hydrophobic surfaces for porous materials like aerogels.

Recently, the use of a combination of polyvinyl alcohol and montmorillonite in aerogels was reported to possess facile preparation, excellent mechanical properties, low thermal conductivity, and fire safety [35,36]. In this work, a simple, convenient, controllable, and efficient templating method was utilized in building a highly hydrophobic surface for aerogels from freeze-drying. It did not require any extra nano–micro additions. By just using sandpaper in the process of freezing, a stable and controllable rough morphology was constructed, which obviously improved hydrophobicity. Polyvinyl alcohol and montmorillonite were chosen to make up an organic–inorganic hybrid framework to ensure the dimensional stability and strength of the samples. After that, methyltrichlorosilane (MTCS) was used to turn the hydrophilic surface into a hydrophobic one. Compared with the no-templating control, the improved aerogel had a higher hydrophobic surface and better water resistance. This novel improvement method for constructing hydrophobic surfaces could increase the possibility of the commercialization of aerogels and provide a reference for the research and development of cheap hydrophobic aerogels.

## 2. Results and Discussion

**Scheme 1:**
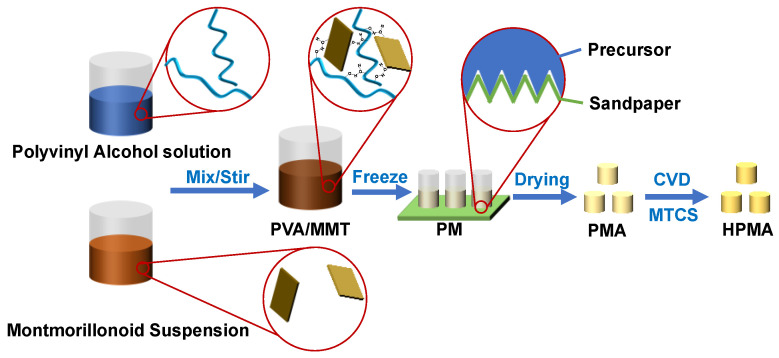
The preparation of HPMAs via a freeze-drying and vapor chemical deposition method.

The preparation processes of the aerogel are illustrated in Scheme 1. The polyvinyl alcohol particles were stirred and dissolved in hot water, and the montmorillonite was dispersed in water by vigorous stirring. The above-mentioned solution and suspension were mixed to prepare the precursor, called PM. During this process, the hydrogen bonds between hydroxyls of PVA and MMT were formed, increasing the viscosity of the mixture significantly. Then, the precursor was poured into the mold, whose inner and bottom were covered with sandpaper, and the mixture wetted and adhered to the rough surface of the sandpaper. After that, a metal plate was framed on the liquid nitrogen, and the molds with precursor were placed on it. The mixture was frozen in this environment, and the surface of ice overlaid and reproduced the morphology of the sandpaper. The aerogels with rough surfaces were obtained by drying them in a lyophilizer, and were called PMAs. Finally, the highly hydrophobic aerogels were obtained by depositing a layer of silane on the skeleton, and were called HPMAs.

**Figure 1:**
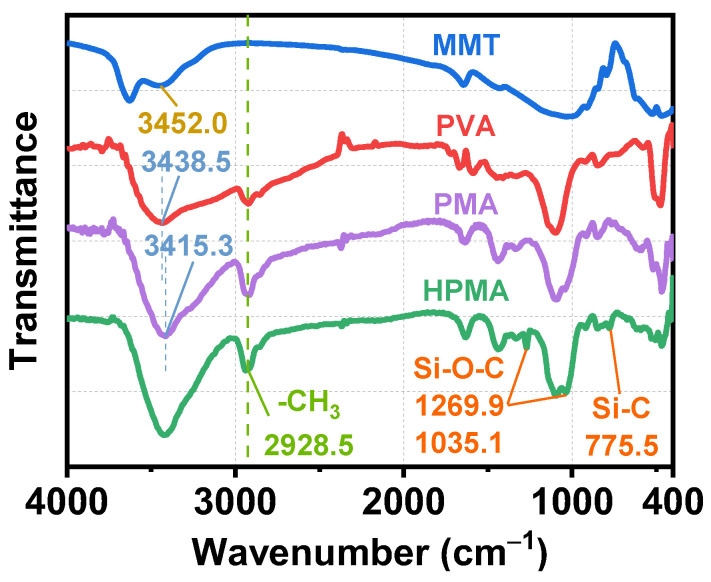
The FTIR spectra of the MMT, PMA, and HPMA.

When the polyvinyl alcohol dissolved, the intramolecular and intermolecular hydrogen bonds were opened, which exposed a large number of hydroxyl groups. It is worth noting that the surface of the MMT layers also contained hydroxyl. This can be proved using Figure 1, where a peak representing hydroxyl stretching vibration is exhibited in the spectra of MMT at 3452.0 cm^−1^ [37]. As the mixture was stirred and aged, the hydroxyl groups of the two materials formed new hydrogen bonds with each other (Scheme 1). The redshift of the peaks representing hydroxyl in the PMA and HPMA (from 3438.5 cm^−1^ to 3415.3 cm^−1^) proves the above phenomenon. Hydrogen bonding enhanced the interaction between organic PVA and inorganic MMT, resulting in better mechanical properties of the material skeleton [38]. This would be beneficial for enabling aerogels to maintain their morphology after leaving the template and would improve the integrated mechanical properties of aerogels. In addition, there were several typical absorption peaks in the infrared spectrum of the pure PVA aerogel. The peaks belonging to the C-C stretching vibration were exhibited at 852.4 cm^−1^ and 1139.7 cm^−1^ (acromion). The peaks corresponding to the C-O stretching vibration, -CH_2_- bending vibration, and C-H stretching vibration were located at 1097.3 cm^−1^, 1440.5 cm^−1^, and 2928.5 cm^−1^. Except for the C-H stretching vibration peak, other absorption peaks did not show significant changes when PVA was blended with MMT or modified by hydrophobicity, indicating the stability of the matrix during the preparation process. This is beneficial for maintaining the stability of the aerogel’s mechanical properties.

**Figure 2:**
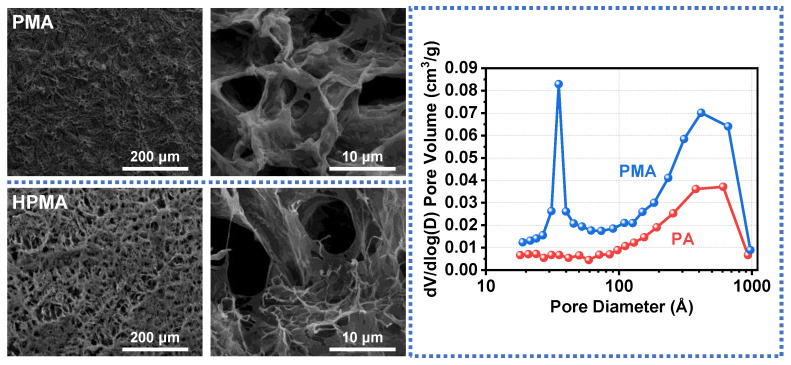
SEM images of the surfaces of PMA before and after hydrophobic modification (named HPMA) (**left**). Pore size distribution of PMA and PVA aerogels (named PAs) (**right**).

The skeleton structure of aerogels has great influence on the properties of materials. As shown by electron microscopy in Figure 2, the morphology of the bottom surface of the PMA was porous, uniform, and relatively flat, on which many slight protrusions caused by uneven shrinkage of the skeleton were distributed. At a smaller observational scale, the PMA had a co-continuous network porous structure, with a pore diameter and skeleton size of about 5~20 μm. Generally, the pore size was dependent on the size of the ice crystal in the freezing process, which is called the ice template method. Benefiting from the use of liquid nitrogen as a cold source, the nucleation rate of liquid was much higher than that under a compression refrigerator, which generated smaller and denser ice crystals in the precursor. Finally, after the sublimation of ice crystals, the obtained PMA had a smaller pore size and finer structure. Because of the strong interaction between PVA and MMT, the MMT sheet and PVA chain were formed as a spatial network structure. This made the viscosity of the precursor much higher than that of the PVA solution or MMT suspension, which appeared as a slurry. During the process of freezing, highly viscous precursors brought great resistance to the growth of ice crystals, making the shape of ice crystals more distorted with more branching. This caused a more complicated pore structure and skeleton structure. As a result, dense secondary constructions were built as “bridges” among the skeletons of aerogels. The effect of the MMT addition on the pore structure can also be observed from the result of the nitrogen adsorption test, where the apparent surface area of the PMA was 22.47 m^2^/g, which was higher than that of PA (10.20 m^2^/g). Moreover, there were pores whose size was ~3.5 nm in the PMA but not in the PA (Figure 2). They may belong to the pores present in the layered structure of montmorillonite. Due to the small pore sizes of these mesopores, their impact on the mechanical strength and hydrophobicity of the material was minimal. These sturdy secondary constructions may enhance the main skeleton of aerogels, enabling the material to have good compression performance [39].

Generally, as density decreases, both the modulus and compressive strength will decrease but the maximum compressive strain will increase. However, a too-high density will not only make the sample difficult to dry but will also make the internal frameworks hard to deposit with silane. The investigation in this article was about the relationship between template roughness and hydrophobicity. So, the density of the PM aerogel (PMA) after freeze-drying was tailored to 0.097 g/cm^3^. The mechanical properties of PMAs are shown in Table 1. The modulus of the PVA was just 2.9 MPa, while the modulus of the PMA increased to 5.5 MPa, proving the enhancement effect of MMT on the PVA skeleton. Although the density and modulus of the PMA are inferior to some aerogels made from two-dimensional materials [40,41,42,43], its mechanical properties are still compared favorably with clay–polymer composite aerogels [44,45]. This ensures that the PMA is not easily deformed or damaged during normal use [46]. It is worth noting that the shape of the skeleton was thin with wrinkles, which was caused by the squeezing between ice crystals during the freezing process. These wrinkles also provided roughness on a micro–nano scale.

**Figure 3:**
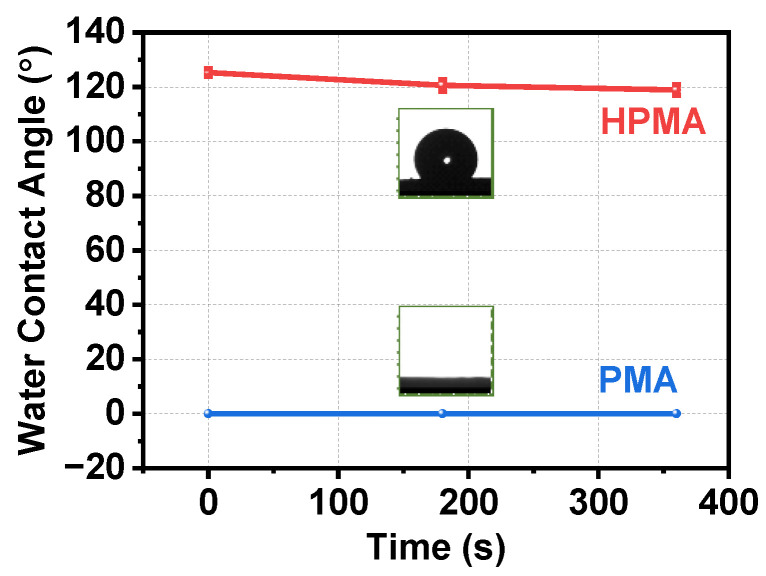
Images of water contact angle measurements of HPMA.

To change the hydrophilicity of the skeleton surface to hydrophobic, MTCS was grafted onto the surface of the skeleton by vapor chemical deposition. The spectral evidence for this is shown in Figure 1. The peaks at 1035.1 and 1269.9 cm^−1^ were attributed to the stretching and bending vibration of the Si-O-C bond, while the peaks at 2928.5 and 775.5 cm^−1^ represented the stretching vibration of the -CH_3_ bond and the Si-C Si-O-C backbone, respectively. During the CVD method, hydrophilic groups on the surface of the skeleton were covered by hydrophobic methyl groups, which made the surface of the aerogel turn from hydrophilic to hydrophobic (Figure 3). The surface of the PMA was nearly super hydrophilic; on it, the water droplet was quickly absorbed by porous structures. After hydrophobic modification, the water contact angle (WCA) of the aerogel increased to 125.3° from about 0°. The change in morphology before and after CVD treatment was investigated by SEM (Figure 2). After the PMA was treated with methyl trichlorosilane by CVD, filamentous substances appeared on the surface of its skeleton. They were formed by the continuous accumulation of silane on the surface [37]. These silane filaments did not change the pore structure of the aerogel, but made the pore wall rough, which was beneficial to its hydrophobic performance. Moreover, the silane covered the hydrophilic groups on the surface of the pores, improving the water resistance of the material. The result of this was that the hydrophobicity of the HPMA was inherent. As shown in Figure 3 and Table 2, the water contact angle of the HPMA decreased no more than 4° over the course of 6 min in an environment with water. Aerogel materials are often exposed to water for a long time when used as insulation or adsorption materials. The coated hydrophobic layer has a poor affinity with the polar aerogel skeleton. The poor interaction often results in the exfoliation or laminar shell-off of the coating while in an environment with water. However, treatment with methyltrichlorosilane was a chemical modification. The hydrophobic silane layer chemically bonded to the hydroxyl groups of the skeleton, fixing the layers firmly to the surface of the skeleton. Furthermore, the silane coating is a polymer material with good molecular chain flexibility at room temperature. This means the silane coating was difficult to crack or shatter. Even though the skeleton expanded due to absorbing water vapor from the air, the silane coating stayed adhered to the skeleton. It is noteworthy that a portion of silane adhered to the surface of the skeleton in the form of fine filaments (Figure 2). These silane filaments overlapped with each other to form a loose spatial network structure, making the water droplet harder to directly contact the skeleton. This “new porous layer” was beneficial for enhancing the firmness of the hydrophobicity on the skeleton surface.

The pore size of the aerogel had no obvious change after hydrophobic treatment (Figure 2). The HPMA still had a uniform porous structure with a diameter of ~10 μm. Using thermal conductivity testing, the thermal conductivity of the material was only 0.055 W/(m·K), proving that it is a qualified insulation material [47]. In general, assuming no coupling of the heat transfer modes, the thermal conductivity (k) of porous materials can be considered the sum of four contributors: k = k_g, cond_ + k_g, conv_ + k_s, cond_ + k_rad_, where k_g, cond_; k_g, conv_; k_s, cond_; and k_rad_ are the thermal conductivity factors for gas conduction, gas convection, solid conduction, and radiation, respectively. Pores with a size lower than 3 mm were considered to have no gas convection (k_g, conv_), which made the total thermal conductivity strongly dependent on gas conduction, solid conduction, and radiation. The pores of the HPMA were larger than 1 μm, which was considered to have no Knudsen effect on gas conduction. Meanwhile, the low density provided almost no advantage in reducing radiation. Therefore, the reduction of thermal conductivity was mainly attributed to solid conduction, due to the fact that the tortuous skeleton structure and rough surface morphology increased the distance of the thermal transportation pathway [1,30,48].

**Figure 4:**
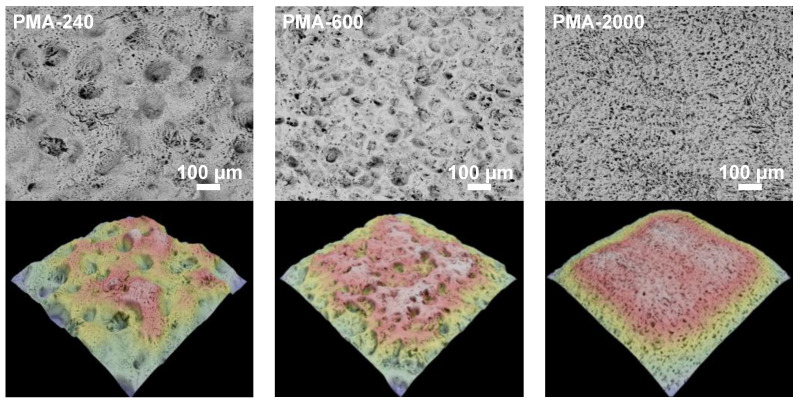
A 3D roughness reconstruction of PMAs treated with different sandpapers.

**Figure 5:**
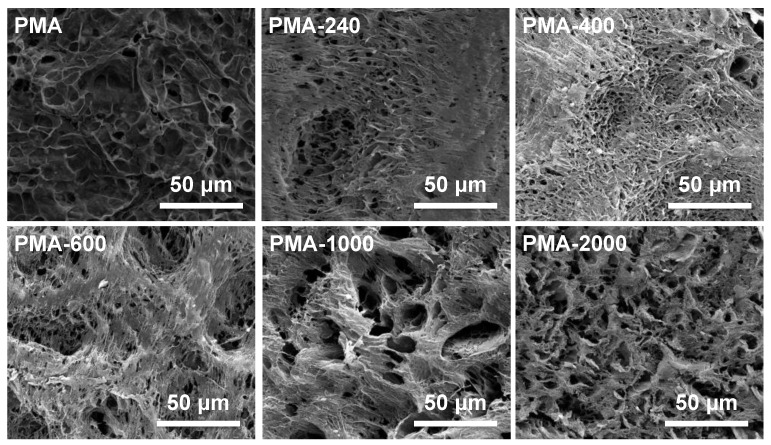
SEM images of the surfaces for PMAs treated with different sandpaper.

During the real use of aerogel materials, if water drops enter the porous structure of an aerogel, the thermal insulation performance of the materials will be affected. What is worse, if the water droplets can wet the skeleton surface to a certain extent, the skeleton could be damaged by the tension of the water droplets, making the aerogel structure collapse. To avoid the occurrence of the above phenomenon, it is necessary to improve the macroscopic hydrophobicity of the material. To this end, a series of aerogels were prepared by using a series of sandpapers with different mesh numbers as templates. By scanning the surface of the sample (Figure 4), it can be seen that the aerogel prepared with a 240-mesh sandpaper as the template was uneven in height distribution and had large fluctuations. There were many pits with a diameter of about 100 μm distributed on the surface. In general, the structural size of the sandpaper with 240 mesh was nearly 60 μm, larger than the size of the pore on PMA-240. It may have been caused by skeleton shrinkage after it dried. The size of the convex part on the surface of the aerogel became smaller, leading to an increase in the size of the holes nearby. However, the surface of PMA-240 still had many pores in the nanoscale which were produced by the ice crystal formation on the surface of the sandpaper. These pores in the nanoscale also contributed to the surface roughness. With the increase in mesh number, the diameter and depth of the pits decreased, and the surface became more regular, which made the size of the structure gradually change from the microscale towards the nanoscale. On the surface of PMA-600, the size of the pits decreased by ~50 μm and they were still larger than the structural size of sandpaper with 600 mesh (58 μm). On the other hand, on the surface of PMA-2000, the size of the pits decreased by 5~8 μm (Figure 5) and they were close to the structural size of sandpaper with 2000 mesh (6.5 μm). This indicates that the higher the mesh of the sandpaper used as a template, the better the adherence of the obtained sample to the surface morphology of the template. The reason for this phenomenon is that the linear shrinkage rate of the aerogel skeleton is almost fixed during freeze drying. Therefore, templates with smaller structural dimensions have smaller size differences in the resulting samples.

Because the mesh number of sandpaper was higher than 240 and its surface morphology size has reached the micro–nano level, an electron microscope was used to further investigate the casting condition of the aerogel surface (Figure 5). The surface of the aerogel with no sandpaper had a porous structure but the upper edge of the pore wall was smooth. Its surface was generally flat without significant protrusions. When templated with sandpaper, the evenness of the pore upper edge obviously decreased. It is worth noting that the size of the secondary pore structures decreased with the increase in mesh number. This is because the size of ice crystals generated on the surface of the sandpaper was limited by the surface structure of sandpaper. The sandpaper with a larger mesh number had a smaller structural size on its surface, limiting the growth of ice crystals in the surface gaps. Consequently, the uneven morphology prevented the generation of ice crystals beyond the structural size on the surface of the sandpaper, resulting in smaller crystal sizes on the surface of the sandpaper with a larger mesh number. Moreover, the smoothness of the edge of the pore wall also decreased with the increase in the sandpaper mesh number. This was due to how, as the surface size of the sandpaper decreased, the wetting effect of the precursor solution on the sandpaper decreased, leaving small bubbles in some of the gaps of the sandpaper. These small bubbles made the surface morphology of the sample incomplete and left a rough edge.

**Figure 6:**
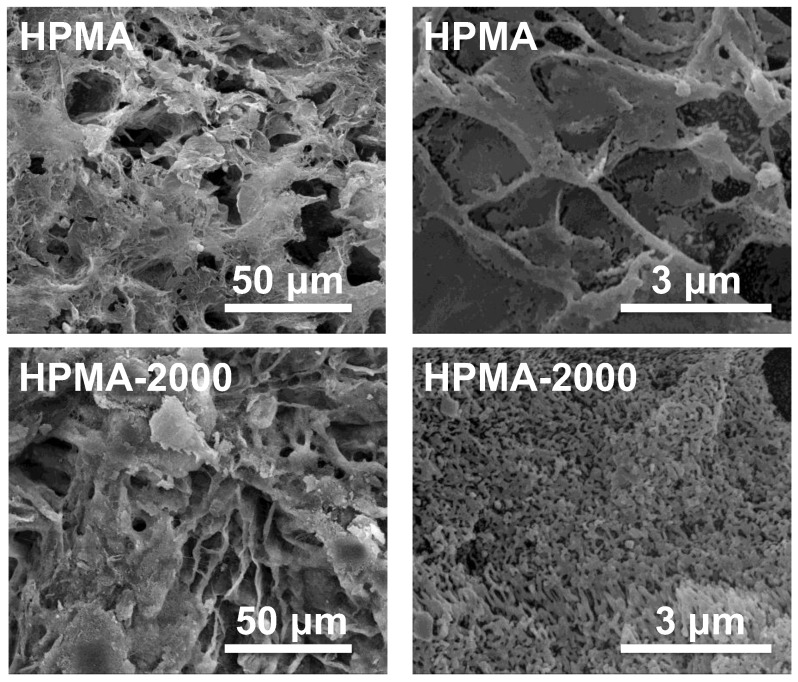
SEM images of the internal cross-section for HPMA and HPMA-2000.

The internal cross-section for HPMA and HPMA-2000 is shown in Figure 6. The pore size inside the aerogels was nearly 50 μm, which was larger than that on the surface. This is because the temperature inside the precursor was higher than that on the bottom, meaning the inside formed bigger ice crystals. The sandpaper templating method only affected the surface of the aerogels, but the inner freezing process and the inner cross-section of HPMA and HPMA-2000 had similar morphologies. At a larger magnification, it could be seen that the internal skeleton surface was also covered with a layer of fine filaments composed of silane. This indicated that MTCS vapor had sufficient penetration ability to fully modify the interior of the porous structure during the CVD process. Furthermore, the internal skeleton had the same hydrophobic layer and hydrophobic properties as the surface skeleton. Aerogels are considered to have a large apparent surface area, so the complicated skeleton inside the aerogel makes it easy to absorb water vapor in the atmosphere. This usually leads to issues such as the thermal insulation performance decreasing, structural shrinkage, and collapse [39,49]. Sufficient treatment of the internal skeleton surface with silane can cover the hydrophilic groups on the internal skeleton, greatly reducing the water absorption performance of the aerogel and ensuring the performance stability of the aerogel. This consistent hydrophobicity inside and outside ensures that even if the sample absorbed water droplets by external forces, the water droplets would not wet the internal skeleton, thus avoiding the sample from being damaged by the capillary force of water droplets.

**Figure 7:**
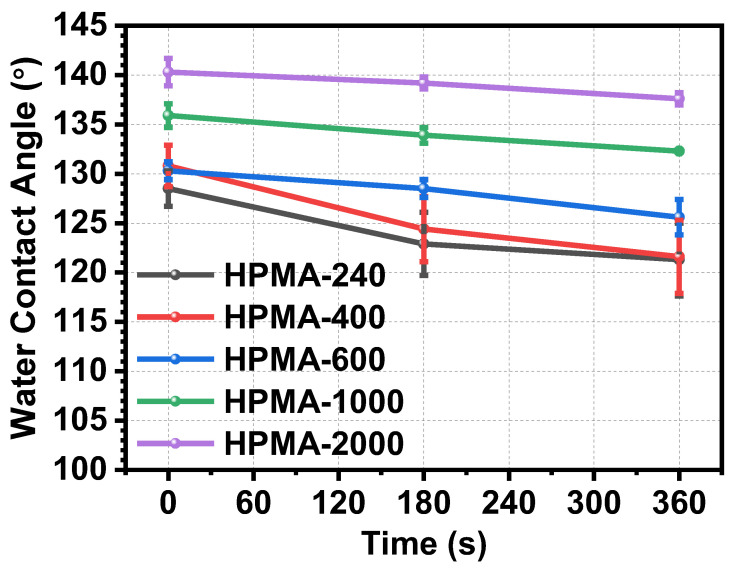
Images of water contact angle measurements of HPMA with sandpaper templating.

According to the Wenzel model, the roughness of a surface affects its water contact angle. Compared with the water contact angle of PMAs without sandpaper templates, the PMAs with different sandpaper templates all have a higher water contact angle (Figure 3 and Figure 7). As shown in Figure 7 and Table 3, the water contact angle of the HPMA increased as the mesh number of templates increased. The highest contact angle of the material was 128.5° with a mesh number of 240 as a template. This was close to the 125.3° of HPMAs without a sandpaper template. While the highest contact angle could reach 140.3° with a mesh number of 2000, this was nearly 15° higher than the contact angle of HPMAs without a sandpaper template. Benefiting from the excellent coating firmness and the hydrophobic stability of the samples, the decrease in the water contact angle did not exceed 10° within 6 min for each sample with a template mesh number of 240 or more. Moreover, in the experiment, it was found that the aerogels prepared in different batches with the same mesh number of sandpaper had similar surface morphologies and water contact angles. This indicated that this method of using sandpaper as a template had good production stability. That is, there was good correspondence between the improvement of the material’s contact angle and the mesh number of sandpaper templating. Therefore, by changing the mesh number of sandpaper, controllable adjustment of the surface water contact angle or roughness of the material can be achieved. When porous materials are used to build thermal insulation layers, they are sometimes not placed in the outermost or innermost layers, but instead act as thermal insulation interlayers that fit together with other materials. A low roughness of the material surface will reduce the interaction force between different layers, while a high roughness will reduce the effective interaction area between layers [49]. Therefore, when thermal insulation aerogels need to fit tightly with other materials, it is necessary to regulate the surface roughness of the aerogels to meet these requirements. This method of using templates to change the roughness can form the desired rough structure in situ during the aerogel preparation process. This eliminates the material post-processing steps before material use, not only reducing the cost of material use but also avoiding the risk of performance degradation which may arise from roughening the aerogel.

## 3. Conclusions

In conclusion, we prepared a porous, strong, thermal insulated, and hydrophobic aerogel from montmorillonite and polyvinyl alcohol. Using sandpaper as the template, rough morphology was successfully prepared on the surface of aerogel, which enabled the high hydrophobicity of the material, and the highest hydrophobicity angle could be increased to 140.3°. Additionally, the hydrophobicity of the material surface could be controlled by changing the mesh number of the sandpaper, and the water contact angle of the material could be varied between 128.5° and 140.3°, which facilitated control of the surface hydrophobicity of the aerogel. Using FTIR analysis, it was found that hydrogen bonds formed between MMT and PVA, which made the skeleton of the aerogel have an excellent mechanical structure compared with the same type of aerogel. This not only ensured that the morphology retained roughness after the templating method, but also endowed the integrity of the aerogel with a high compression strength (with a mechanical strength of 1.25 MPa at 50% compression). After being grafted with methyltrichlorosilane by chemical vapor deposition, hydrophilic groups on the surface of the skeleton were covered by hydrophobic methyl groups, which endowed the aerogel with the ability to tolerate water for a long time. Due to its micro-size and uniform porous structure, the obtained aerogel had high thermal insulation properties with low thermal conductivity. It was also found that the addition of MMT could improve the apparent surface area of the aerogel. This aerogel with excellent comprehensive performance was successfully prepared by this simple and convenient templating method, which lays the foundation for the wide use and commercialization of aerogels.

## 4. Materials and Methods

### 4.1. Preparation of PVA/MMT Precursor

A total of 10 g of polyvinyl alcohol was added to 90 g of deionized water and dissolved by stirring the mixture intensely at 90 °C, called Solution A. Meanwhile, the MMT particles were added to deionized water in a mass fraction of 10 wt% and dispersed by high-speed (10,000 rpm) stirring, called Dispersion B. Then, the PVA/MMT precursor was obtained by stirring equal masses of Solution A and Dispersion B until a uniform and stable dispersion was formed.

### 4.2. Preparation of Aerogels by Template

Sandpapers with different mesh numbers were used as the template to control and change the roughness of the aerogel surface. Sandpaper was placed on the bottom and inner wall of a circular or square through-hole mold, and then the prepared precursor was poured into the molds. All the molds were frozen by placing them on a steel plate which was immersed in liquid nitrogen. Then, the frozen precursor with molds was placed in the lyophilizer and freeze-dried at −20 °C for 72 h to obtain the aerogels. The prepared aerogels were named PMA-xxx, whose mesh numbers of the sandpaper template were xxx.

### 4.3. Preparation of Hydrophobic Aerogels

The hydrophobic aerogels were prepared by chemical vapor deposition with methyltrichlorosilane. The untreated aerogels and a small vessel with 1 mL methyltrichlorosilane were placed in a vacuum desiccator. The desiccator was pumped to a vacuum degree of 0.1 bar and placed in a 50 °C oven for 3 h. Subsequently, the treated aerogels were placed in a vacuum oven with NaOH for 24 h to remove the excess silane and by-products (mainly HCl). The obtained hydrophobic aerogels were called HPMAs.

### 4.4. Characterization

Fourier-transform infrared (FTIR) spectra were obtained by a Nicolet 6700 spectrometer (Thermo Fisher, Waltham, USA). The morphology was characterized by scanning electron microscopy (SEM) on a JSM-5900LV (JEOL, Tokyo, Japan) equipped with energy-dispersive X-ray spectroscopy (EDX). Water contact angles (WCAs) were measured by a JC2000D2H contact angle goniometer (POWEREACH, Shanghai, China). The mechanical properties were investigated by a universal testing machine (Instron 5400, Boston, MA, USA) equipped with a 500 N load. Surface 3D roughness was characterized by an Aphox scanning cine instrument and its accessories (Phenom-world, Eindhoven, The Netherlands). The nitrogen adsorption test was conducted by an ASAP 2420 (Micromeritics, Norcross, GA, USA).

## Figures and Tables

**Table 1:** Densities and mechanical properties for the resulting aerogels.

Sample	Modulus (MPa)	Density (g/cm^3^)	Specific Modulus (×10^3^ m)
PVA	2.9 ± 0.5	0.0754 ± 0.003	~38.6
PMA	5.5 ± 0.4	0.0972 ± 0.0004	~56.6

**Table 2:** WCA tests data of HPMA.

Sample	t = 0 s	t = 180 s	t = 360 s
PMA	0°	0°	0°
HPMA	125.3 ± 1.4°	120.6 ± 2.1°	121.7 ± 3.7°

**Table 3:** The WCA test data of HPMAs with sandpaper templating.

Sample	t = 0 s	t = 180 s	t = 360 s
HPMA-240	128.5 ± 1.8°	122.9 ± 3.2°	121.3 ± 3.7°
HPMA-400	130.8 ± 2.1°	124.4 ± 3.3°	121.7 ± 3.7°
HPMA-600	130.3 ± 0.9°	128.5 ± 0.9°	125.6 ± 1.8°
HPMA-1000	135.9 ± 1.2°	133.9 ± 0.8°	132.3 ± 0.1°
HPMA-2000	140.3 ± 1.4°	139.2 ± 0.6°	137.6 ± 0.6°

## Data Availability

Data are contained within the article.
